# MetaboScope: a statistical toolbox for analyzing ^1^H nuclear magnetic resonance spectra from human clinical studies

**DOI:** 10.1093/bioadv/vbae142

**Published:** 2024-10-28

**Authors:** Ruey Leng Loo, Javier Osorio Mosquera, Michael Zasso, Jacqueline Mathews, Desmond G Johnston, Jeremy K Nicholson, Luc Patiny, Elaine Holmes, Julien Wist

**Affiliations:** Australian National Phenome Centre and Centre for Computational and Systems Medicine, Health Futures Institute, Murdoch University, Perth, WA 6150, Australia; Australian National Phenome Centre and Centre for Computational and Systems Medicine, Health Futures Institute, Murdoch University, Perth, WA 6150, Australia; Chemistry Department, Universidad del Valle, Cali 76001, Colombia; Zakodium Sàrl, Lonay 1027, Switzerland; NIHR CRN Specialty Cluster A, Department of Gene Therapy NHLI, Imperial College, London SW3 6LR, United Kingdom; Department of Metabolism Digestion and Reproduction, Faculty of Medicine, Imperial College London, London SW7 2AZ, United Kingdom; Australian National Phenome Centre and Centre for Computational and Systems Medicine, Health Futures Institute, Murdoch University, Perth, WA 6150, Australia; Department of Surgery and Cancer, Institute of Global Health and Innovation, Imperial College London, London, SW7 2AZ, United Kingdom; Zakodium Sàrl, Lonay 1027, Switzerland; Australian National Phenome Centre and Centre for Computational and Systems Medicine, Health Futures Institute, Murdoch University, Perth, WA 6150, Australia; Department of Metabolism Digestion and Reproduction, Faculty of Medicine, Imperial College London, London SW7 2AZ, United Kingdom; Australian National Phenome Centre and Centre for Computational and Systems Medicine, Health Futures Institute, Murdoch University, Perth, WA 6150, Australia; Chemistry Department, Universidad del Valle, Cali 76001, Colombia; Department of Metabolism Digestion and Reproduction, Faculty of Medicine, Imperial College London, London SW7 2AZ, United Kingdom

## Abstract

**Motivation:**

Metabolic phenotyping, using high-resolution spectroscopic molecular fingerprints of biological samples, has demonstrated diagnostic, prognostic, and mechanistic value in clinical studies. However, clinical translation is hindered by the lack of viable workflows and challenges in converting spectral data into usable information.

**Results:**

MetaboScope is an analytical and statistical workflow for learning, designing and analyzing clinically relevant ^1^H nuclear magnetic resonance data. It features modular preprocessing pipelines, multivariate modeling tools including Principal Components Analysis (PCA), Orthogonal-Projection to Latent Structure Discriminant Analysis (OPLS-DA), and biomarker discovery tools (multiblock PCA and statistical spectroscopy). A simulation tool is also provided, allowing users to create synthetic spectra for hypothesis testing and power calculations.

**Availability and implementation:**

MetaboScope is built as a pipeline where each module accepts the output generated by the previous one. This provides flexibility and simplicity of use, while being straightforward to maintain. The system and its libraries were developed in JavaScript and run as a web app; therefore, all the operations are performed on the local computer, circumventing the need to upload data. The MetaboScope tool is available at https://www.cheminfo.org/flavor/metabolomics/index.html. The code is open-source and can be deployed locally if necessary. Module notes, video tutorials, and clinical spectral datasets are provided for modeling.

## 1 Introduction

Molecular phenotyping has emerged as an essential tool in exploring, characterizing, and understanding the dynamic interactions between our genes and environment (diet, lifestyle, microbiomes), and their phenotypic expression across diverse human populations. Analytical platforms for deep phenotyping of biofluids such as plasma, urine and cerebrospinal fluids, based on nuclear magnetic resonance (NMR) spectroscopy and mass spectrometry (MS), can generate metabolic “fingerprints” that contain latent information relating to physiological or pathological status, informing on disease mechanisms, diagnosis and prognosis (Hood and Flores 2012, Sarafian *et al.* 2015, Fatemi *et al.* 2022, Perez-Diaz-Del-Campo *et al.* 2022).

Clinicians are now beginning to adopt metabolic phenotyping as part of a systems medicine approach to managing diseases. However, the technology can be off-putting, and this hinders its routine implementation in the clinical setting. The diversity of analytical platforms and even broader range of operational parameters implemented across those platforms presents a challenge, particularly for the clinician scientist users who lack an analytical chemistry background. For clinicians and biomedical scientists, the ability to visualize complex spectral data in a meaningful way is essential. There are a number of statistical toolboxes and softwares available for analyzing metabolomic data with varying levels of capacity for data handling and visualization such as SIMCA P (Sartorius AG), Metaboanalyst (Pang *et al.* 2021), Unscrambler (AspenTech), PLS-toolbox (Solutions4U) each with different features. Use of automated softwares delivering assigned and quantified data without reference to the NMR spectra has resulted in increasing numbers of publications with incorrect assignments and consequently wrong biological interpretation of results. Some softwares, e.g. NMRProcFlow (Jacob *et al.* 2017) have developed preprocessing pipelines to encourage users to visualize the NMR spectra. Nevertheless, a gap in most of currently available software packages is the ability to interact with well-documented clinical datasets and to learn from more familiar data. In response to this knowledge gap, we have developed a software suite that includes video tutorials, statistical modeling tools, exemplar clinical and model datasets, and data synthesis features to allow the user to become familiar with the technology using real clinical data and to test hypotheses regarding group size and composition.

## 2 MetaboScope

We present an integrated workflow “MetaboScope,” which has been implemented for the analysis of NMR data and has several advantages over other workflows currently available. The workflow is open-source and, although the set of modeling tools are not as extensive as other softwares, the system is highly interactive and focused on the co-analysis of clinical and spectral data. Additionally, the workflow allows input of data from various sources including JCAMP/Bruker/JEOL formats and does not require prior transformation of data.

The software suite is organized as a set of eight modules that can be used incrementally to complete a full metabolic profiling analysis pipeline or to use as stand-alone modules to implement single stages of the pipeline. The eight modules are associated with a set of 11 short videos and three example datasets, with spectra and associated annotations for the user to familiarize themselves with the technology. Detailed rationale for the creating and implementation of the Metaboscope, the exemplar datasets, instructions for installation, module descriptions, video tutorials and a user manual are provided in Supplementary Material.

**Figure 1.**
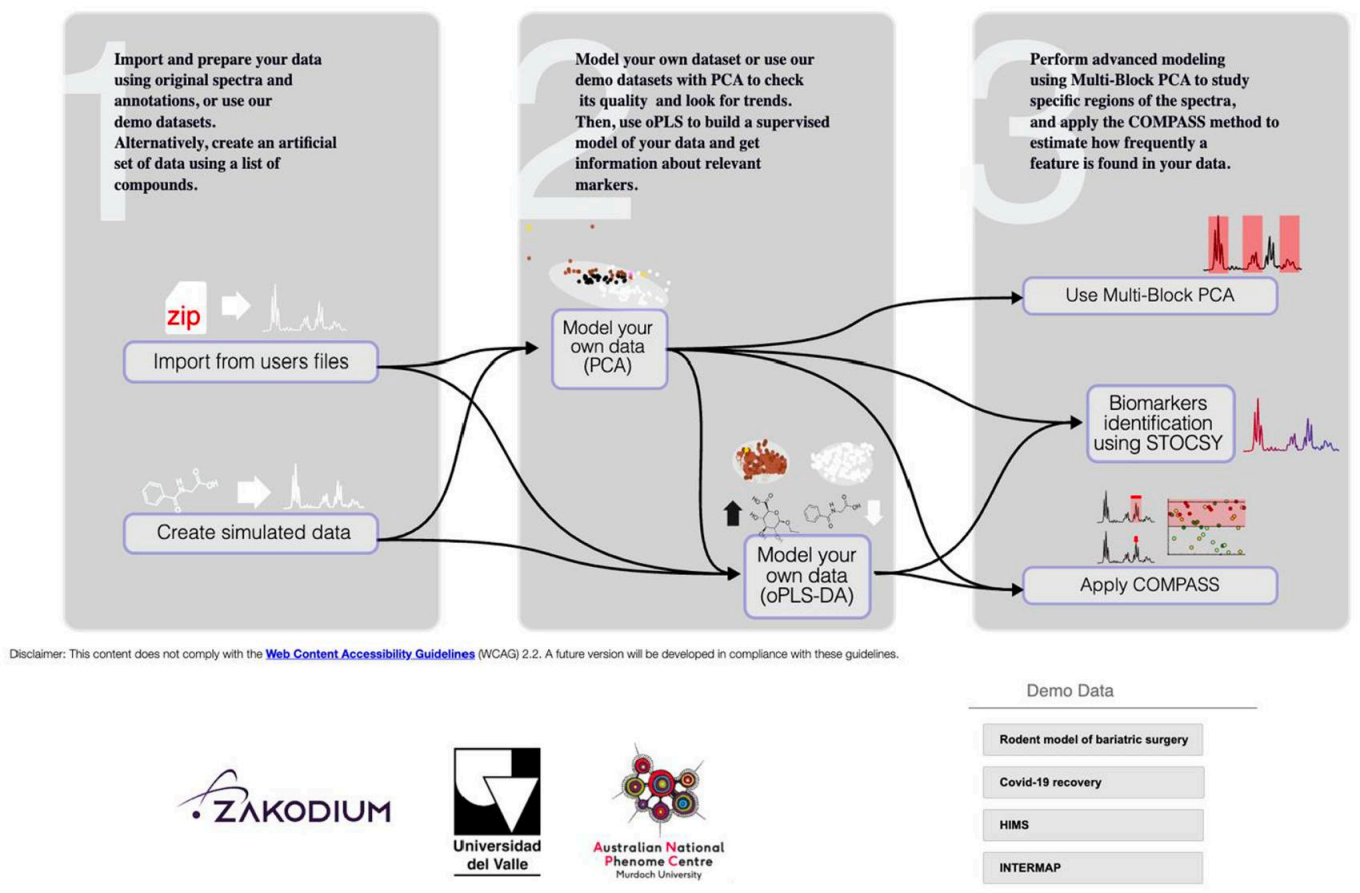
Schematic overview of the MetaboScope software suite.

Video 1: Landing page and uploading data: Presents the overall architecture of the software suite (Fig. 1).

Video 2: Uploading spectral data: Options are provided for the upload of user data, exemplar clinical spectral datasets or simulated datasets together with annotation files containing spectral identifiers, class information and clinical metadata if required. Data files are then exported in a .tsv format prior to performing statistical analysis.

Video 3: Data processing: Provides capacity for removing unwanted spectral regions, scaling functions and normalization of spectra.

Video 4: Principal components analysis (PCA): Interactive function to allow users to identify outliers and recalculate PCA models iteratively.

Module 5: Statistical Correlation SpectroscopY (STOCSY): STOCSY (Cloarec *et al.* 2005) aids the structural identification of signals of interest identified in the loadings plots of multivariate modeling. An additional feature in the data visualization panels is the ability to rank datapoint intensities to establish which metabolites or signals are the most abundant for a given metabolite.

Video 6: Box plot features: Univariate analysis on signals (metabolites) of interest. Available statistics include displaying the min-max range, the interquartile range and the median and allows individual spectra to be mapped onto the ranges to ascertain where each individual sits within their respective group.

Video 7: Orthogonal partial least square discriminant analysis (OPLS-DA): Used to maximize separation between two or more classes and map test sets onto the OPLS-DA model to predict class membership (Bylesjö *et al.* 2006). The outputs display the contributions of metabolites to the predictive and the orthogonal component.

Video 8: Multiblock PCA: Allows the user to model selected subsections of the spectrum with options to scale or preprocess each block individually in order to overcome issues arising from dynamic range problems and provide finer detail of the biologically relevant structure in the dataset (Westerhuis *et al.* 1998).

Video 9: COmbined Multiblock Principal Component Analysis with Statistical Spectroscopy (COMPASS) (Loo *et al.* 2021): Provides an efficient method for identifying specific spectral patterns in a dataset to identify the number of samples containing a particular metabolite. A user-defined threshold based on the cross-correlation values ensures the unambiguous identification of samples containing the reference pattern, which the user defines based on a clear, not overlapped, and high intensity spectrum.

Video 10: COMPASS and STOCSY: Interactive iteration of COMPASS and STOCSY functions to improve the certainty of capturing the true molecular signature of a given metabolite.

Video 11: Generate simulated data: Simulated spectra are created using a list of chemicals defined by either SMILES codes or drawn in a chemical structure drawing panel. The following parameters can be adjusted to explore their impact on spectral data: number of classes; identified “biomarkers”; number of spectra per class; and compound concentration ranges. Once a simulated spectral dataset is created, all the normal functions of the toolbox can then be utilized.

## 3 Suitability of the software suite for biomedical applications

Human and patient data require a high level of data security. Although the workflow operates as an App within a browser framework, it circumvents many data privacy issues by keeping and analyzing data locally on the host computer, thereby making the process faster. Implementing the pipeline in a browser environment also ensures that software updates are immediately accessible and independent of the operating system (e.g. Mac, PC). Our pipeline offers another valuable feature: three clinical datasets are provided along with detailed video tutorials, serving as a training resource for users to build their expertise. The option to create and use simulated data provides an additional resource for evaluating the impact of sample size and composition on the models and subsequent biological inferences drawn.

The lack of standardization across all aspects of the metabolic phenotyping workflow, from experimental design to data analysis, hinders the integration of the technology into standard clinical practice. Additionally, the multivariate data methods required to model and interpret the statistical data are often unfamiliar to those used to handling standard clinical data. The complexity of clinical data necessitates a flexible analytical environment to accommodate idiosyncratic patients, a high genetic and environmental variation in response to disease or therapeutic interventions, and frequent missing data. Thus, in MetaboScope we have emphasized the capacity for interaction with the spectral data and the multivariate models, allowing users to select samples of interest from the PCA/OPLS-DA models and to interrogate their composition with respect to their “fit” with other samples in the class or model. For example, users can select “outliers” and choose to exclude them interactively, recalculating the model while projecting the excluded sample back into the model to evaluate the impact of its removal. Another useful feature for clinical data is the ability to easily interchange between visualizing multivariate and univariate models. For example, based on an OPLS-DA model, users can define several candidate biomarkers for a particular disease condition. The interactive univariate statistics display (min-max range, first and third interquartile, and median) for each spectral datapoint, either on a class-by-class or whole dataset basis, enables users to assess class differences or determine relative concentrations within the cohort. The pipeline’s capability for performing COMPASS and multiblock modeling allows for focusing on particular sets of metabolites or defining patterns within the data that can be identified and automatically ranked, regardless of dataset size. Future versions of MetaboScope are expected to include additional features such as peak integration for absolute quantifications of markers of interests.

## 4 Conclusion

The MetaboScope software suite is an integrated workflow for the analysis of NMR data that is aimed at providing a flexible resource for clinicians and biomedical scientists. Basic unsupervised and multivariate regression functions are provided in a web App with focus on interactive co-modeling of spectral and clinical data. The software suite is organized as a set of modules that can be used incrementally to complete a full metabolic profiling analysis pipeline or to use as stand-alone modules to implement single stages on the pipeline. Exemplar spectral datasets, video tutorials and a detailed user manual are provided to help users navigate the software.

## Supplementary Material

vbae142_Supplementary_Data

## Data Availability

The NMR example datasets are accessible directly via https://www.cheminfo.org/flavor/metabolomics/index.html. For the description of each dataset, please refer to the supplementary information.
