# Supplementary material for: MetaboScope: a statistical toolbox for analyzing 1H nuclear magnetic resonance spectra from human clinical studies
Source: Bioinform Adv. 2024 Oct 28;4(1):vbae142. doi: 10.1093/bioadv/vbae142 (PMC11576352; doi:10.1093/bioadv/vbae142)
Supplement: vbae142_Supplementary_Data [file vbae142_supplementary_data.docx]

**Metaboscope: A statistical toolbox for analyzing ^1^H NMR spectra from human clinical studies**

Ruey Leng Loo^1#^, Javier Osorio Mosquera^1,2#^, Michael Zasso^3^, Jacqueline Mathews^4^, Desmond G Johnston^5^, Jeremy K Nicholson^1,6^, Luc Patiny^3^, Elaine Holmes^1,5*^, Julien Wist^1,2*^

^1^Australian National Phenome Centre and Centre for Computational and Systems Medicine, Health Futures Institute, Murdoch University, Perth, WA 6150, Australia

^2^Chemistry Department, Universidad del Valle, Cali 76001, Colombia

^3^Zakodium Sàrl, Lonay 1027, Switzerland

^4^Department of Gene Therapy NHLI, Imperial College London, Emmanuel Kaye Building, London, SW3 6LR, United Kingdom

^5^Department of Metabolism Digestion and Reproduction, Faculty of Medicine, Imperial College London, London SW7 2AZ, United Kingdom

^6^Department of Surgery and Cancer, Institute of Global Health and Innovation, Imperial College London, London, SW7 2AZ, United Kingdom

*** Correspondence to: Address:** Australian National Phenome Centre and Centre for Computational and Systems Medicine, Health Futures Institute, Murdoch University, 5 Robin Warren Street, Murdoch, WA 6150, Australia. Email: **Julien.wist@murdoch.edu.au,** [**elaine.holmes@imperial.ac.uk**](mailto:elaine.holmes@imperial.ac.uk)

^#^ These authors contributed equally to the publication.

**Content**

1.1 The rationale for the creation and implementation of Metaboscope

1.2 Description of example datasets

1.3 Overview of Metaboscope

1.4 Benefits and limitations of the software

1.5 Instruction for installation

1.6 Eleven short modules corresponding the videos:

Module 1: Landing page and uploading data (https://youtu.be/QTWl7wxEYSM)

Module 2: Uploading spectral data (https://youtu.be/gL7cSiDcIYk)

Module 3: Data processing (https://youtu.be/13YV7DeFZ6s)

Module 4: Principal components analysis (PCA) (https://youtu.be/FeHvPYGg8Kk)

Module 5: Features for visualization (https://youtu.be/7xhyQwchH_k)

Module 6: Features in the visualization II: Box plot (https://youtu.be/PVxTac7w9Gk)

Module 7: Orthogonal Projection to Latent Structure Discriminant Analysis (OPLS-DA) (https://youtu.be/kyvTJNfBNT4)

Module 8: Multiblock PCA (https://youtu.be/aJSAlOyYtJc)

Module 9: COmbined Multi-block Principal component Analysis with Statistical Spectroscopy (COMPASS) (https://youtu.be/dm1i3ASMv3Q)

Module 10: COMPASS and STOCSY (<https://youtu.be/MaurOIipPKA>)

Module 11: Generating simulated datasets

**1.1 The rationale for the creation and implementation of Metaboscope**

The emergence of Precision Medicine is revolutionizing how we think about stratifying patients for human diseases as we respond to a changing socioeconomic landscape. The advantage of stratifying patients with respect to disease management is twofold: firstly, it facilitates the selection of appropriate drugs or other therapies at the earliest possible time point in the patient journey, thereby directly benefiting the patient; Secondly, by optimizing the selection of ‘the right’ drug, it reduces unnecessary expenditure and therefore has a beneficial economic impact. Recent publications have concluded that diagnostic tests in the future will be based on measuring numerous markers, whose interpretation utilizes mathematical algorithms to identify signatures characteristic of disease stage and type (Fatemi, et al., 2022; Hood and Flores, 2012; Perez-Diaz-Del-Campo, et al., 2022).

Driven by this need, molecular phenotyping has emerged as an essential tool in exploring, characterizing, and understanding the dynamic interactions between our genes and environment (diet, lifestyle, microbiomes), and their phenotypic expression across diverse human populations. Analytical platforms for deep phenotyping of biofluids such as plasma, urine, and cerebrospinal fluid based on nuclear magnetic resonance (NMR) spectroscopy and mass spectrometry (MS) can generate metabolic “fingerprints” that contain latent information relating to physiological or pathological status, informing on disease mechanisms, diagnosis and prognosis (Sarafian, et al., 2015). This technology provides an efficient tool for biomarker discovery, evaluating clinical management of disease, and for interrogating mechanisms of disease thereby opening new opportunities in personalized healthcare for mapping patient journeys and patient stratification.

Clinicians are now beginning to adopt metabolic phenotyping as part of a systems medicine approach to managing diseases. Similarly, the use of multi-omics strategies to gain a deeper understanding of disease processes is gaining popularity. However, analytical chemical technology can be off-putting and this can hinder its use in the clinical setting. The diversity of analytical platforms (liquid chromatography-mass spectrometry (LC-MS); gas chromatography-mass spectrometry (GC-MS); direct infusion MS, infrared spectrometry, etc) and the even broader range of operational parameters implemented across those platforms presents a challenge, particularly for clinician-scientist who lack an analytical chemistry background. Lack of standardization across all aspects of the workflow, from experimental design to data analysis, further compounds the integration of the technology into standard clinical practice and multivariate data methods required to model and interpret the statistical data are often unfamiliar to those used to handling standard clinical data. Above all, the ability to visualize complex spectral data in a meaningful way is essential. There are a number of statistical toolboxes and software packages available for analyzing metabolomic data with varying levels of capacity for data handling and visualization such as SIMCA® (<https://www.sartorius.com/en/products/process-analytical-technology/data-analytics-software/mvda-software/simca>), Metaboanalyst (https://www.metaboanalyst.ca), Unscrambler ([www.camo.com/unscrambler/](http://www.camo.com/unscrambler/)) and PLS-toolbox (https://www.solutions4u-asia.com/pdt/EV/S4U-EV-PLS_Toolbox.html) each with different features. However, a gap in most of these software packages is the ability to interact with well-documented clinical datasets and to learn from more familiar data.

In response to this market gap, we have developed a software suite complete with video tutorials, user manual, statistical modeling tools, exemplar clinical and model data sets, and data synthesis features to allow the user to test hypotheses regarding group size and composition. This software suite, ‘Metaboscope’, is aimed at encouraging the use of metabolic phenotyping in clinical studies. It is designed for clinicians, health workers, and basic scientists who would like to use the technology appropriately without needing to become experts in specific analytical or bioinformatic domains. The exemplar datasets with worked examples allow users to familiarise themselves with the various features in the workflow prior to importing and modeling their own datasets. The first version of the software suite is dedicated to the analysis of NMR spectroscopic datasets with the scope to expand to accommodate data from other analytical platforms.

**1.2 Description of example datasets:**

1) **Rodent model of bariatric surgery.**

This dataset is described in full in (Li, et al., 2011). Briefly, it comprises two groups of rats (n=6 per group), which have undergone either Roux-en-Y gastric bypass (RYGB) surgery or sham surgery, to replicate the impact of surgical stress. Urine samples were collected pre-surgery and at three time points post-surgery (2, 6 and 8 weeks). A ^1^H NMR spectrum was acquired for each urine sample and data are made available in “bariatric surgery: rat model”. Although the tool provided is primarily designed for use with human clinical datasets, the worked example using the rat model of bariatric surgery demonstrates an unambiguous surgery-related profile and is not compromised by the genetic and environmental variation encountered in human studies. The time of sampling is provided so that users can explore the evolving metabolic impact of bariatric surgery over time. Practicing modeling of metabolomic data using a model dataset with relevance to the human condition has the advantage of providing clear solutions as to the phenotypic description of class membership, whilst delivering biologically relevant information since the response to bariatric surgery is well conserved between mouse (Seyfried, et al., 2013), rat (Li, et al., 2011) and human (Li, et al., 2021) models.

2) **COVID-19 recovery.**

This dataset contains ^1^H NMR spectra from a cohort of SARS-CoV-2 infected participants and matched controls. A full description of this dataset is provided in (Holmes, et al., 2021). Plasma samples were obtained from a non-hospitalized group of 27 participants 3 months after being confirmed as being SARS-CoV-2 positive and compared with a group of 18 hospitalized patients in the acute phase of the infection and 41 healthy control participants. Two spectral datasets are provided: i) the standard 1-dimensional dataset containing contributions from macromolecules, such as lipoproteins, as well as smaller molecules such as amino acids and sugars; and ii) a Carr-Purcell Meiboom-Gill (CPMG) spectrum whereby the signals from macromolecules are attenuated in order to better visualize the small molecules. The infection status, age, and sex of participants are provided.

3) **HIMS.**

The Health In Men Study (HIMS) was designed as a population-based randomized trial of screening for abdominal aortic aneurysms conducted in Perth, Western Australia (Norman, et al., 2009). There were 12,203 men aged 65 years or more, who were enrolled in 1996-1999. 4,248 of these men returned for assessment in 2001-04, and 1,434 men were assessed in 2011-12 (Yeap, et al., 2018). From the men assessed in 2011-12, 100 men without diabetes but with a history of myocardial infarction, as well as 100 controls matched for age and BMI ranges with no history of either diabetes or cardiovascular diseases, were selected. Standard 1D and CPMG Plasma spectra, acquired from these participants have been provided along with age and BMI.

4) **INTERMAP**

The INTERMAP Study is an international co-operative epidemiological investigation into macro- and micronutrient intakes and other factors influencing blood pressure levels of individuals (Stamler, et al., 2003). It enrolled men and women ages 40-59 years. The urine samples (based on 24-hour urine collection) selected for this dataset relate to the USA arm of the study from which 150 participants with obesity (body mass index, BMI > 30) and 150 participants with healthy weight (BMI between 18.5 and 25).

**1.3 Overview of Metaboscope:**

The software suite is comprised of three main components that consist of A) a data importation pipeline that allows users to import their own dataset or create a simulation dataset to test hypotheses and facilitate power calculations for defining class sizes; B) basic data modelling using principal components analysis (PCA) and orthogonal projection to latent structure discriminant analysis; and C) advanced data modelling using COmbined Multiblock Principal components Analysis and Statistical Spectroscopy (COMPASS) (Loo, et al., 2021); Statistical TOtal Correlation SpectroscopY (STOCSY) (Cloarec, et al., 2005) tools for identification of candidate biomarkers; and multiblock PCA (Torell, et al., 2020).

**1.4 Benefits and limitations of the software**

1) The data do not require submission to a server and remain on the host computer, thereby circumventing data privacy issues around storing and transferring human data.
2) The pipeline operates in a browser environment. Thus, software updates are immediately accessible and independent of the operating system (e.g. Mac, PC).
3) The workflow is open-source and, although the set of modeling tools is not as extensive as other software, the system is highly interactive and focused on the co-analysis of clinical and spectral data. Thus, clinical data can be updated independently of the spectra and since the annotation and spectral files are separate, updated information can be easily imported and linked to the spectra.
4) The workflow allows input of data from various sources including JCAMP/Bruker/JEOL formats and does not require prior transformation of data.
5) The video modules and user manual provide step-by-step instructions on how to use various features and functions in the software, with functions divided into generating simulated dataset, uploading and pre-processing actual spectroscopic data, data modeling using standard methods, and more advanced data modeling options such as multiblock PCA.
6) Emphasis is placed on the capacity for interaction with the spectral data and the multivariate models. The user can iteratively select samples of interest from the PCA models, for example, class outliers, and assess the effect on the model after the exclusion of the samples. On the other hand, idiosyncratic samples may carry valuable information regarding inter-individual differences in response to a disease or therapeutic intervention. The interactivity of the user interface within the Metaboscope allows the user to identify and explore the spectral profile of samples of interest.
7) The importation of an annotation excel file separately to the spectral data file allows the file to be updated easily as further clinical data are generated and re-imported.
8) Facility for interchanging between visualizing multivariate and univariate models. For example, based on an orthogonal partial least square discriminant analysis (OPLS-DA) model, the user may define several candidate biomarkers for a particular disease condition from the OPLS-DA coefficients. The univariate statistics (min-max range, first and third interquartile, and median) for each spectral datapoint on a class-by-class or whole dataset basis allows the user to assess class differences or to see where a sample of interest lies within the cohort.
9) Multiblock PCA and OPLS-DA tools allow the user to segment spectra into subsections either automatically e.g. into a series of 0.5 ppm spectral windows, which can be modeled independently, or by user-defined regions to focus on specific regions. The advantage of modeling ‘blocks’ of data independently is that high-intensity resonances that may dominate the global spectral profile. Taking type 2 diabetes as an example signals from glucose in diabetic patients may bias the whole model, and therefore dividing the spectrum into smaller sections may allow the influence of metabolites that are present in lower concentrations but are nevertheless significant, such as branched-chain amino acids, to be identified.
10) COMPASS (COmbined Multiblock Principal components Analysis and Statistical Spectroscopy) (Loo, et al., 2021) is also provided as a tool. The clinical utility of COMPASS is to facilitate the detection of all samples in a dataset that contains a particular metabolite based on matching a spectral reference pattern to each spectrum. It may be desirably to identify and subsequently exclude, drug metabolites from the spectral dataset, to know how many patients were taking particular medications, or to calculate how many (and which) samples contained a particular disease-related biomarker.
11) The ability to download .tsv files after each model iteration provides an easy method for version control of models.
12) Both urine and serum/plasma demo datasets, which match examples in published literature to provide a benchmark for users who are learning how to use the software suite.
13) To expedite the user’s familiarity with the software suite, a series of 11 video tutorials are provided using a published bariatric surgery dataset that takes the user through a typical data analysis pipeline in a stepwise manner.
14) When designing a study, one of the most commonly debated issues is how to decide on sample size. It is difficult to calculate the power for multivariate studies since the user does not know how many biomarkers will be relevant at the outset of the experiment and only a few examples exist in the literature e.g.(Blaise, et al., 2016) . The provision of a tool for simulating a ‘mock’ biofluid data set enables the user to specify the number of samples per group, number of metabolites per sample and the number and concentration range of putative biomarkers. The simulated dataset can then be processed and modelled, adjusting the parameters to ascertain the impact of changing those parameters.

**1.5 Instruction for installation**

Note: Users can access all necessary functions without performing this installation at <https://www.cheminfo.org/flavor/metabolomics/index.html>

Step 1. Install roc-eln-docker

The MetaboScope tools are deployed over the ELN system developed by Cheminfo. To enable the tools described in this paper to run locally on your computer, you must first install the ELN system by following the instructions at <https://github.com/cheminfo/roc-eln-docker>.

Further, a detailed explanation about the configuration of the ELN and its views is available at <https://docs.c6h6.org/docs/eln/installation/customization>

Step2: Clone the Metaboscope views on the ELN system

2.1. Open the on-tabs manager at http://localhost/on-tabs.

Note: We assume the main application is running at <http://localhost/> and you must log in as the system admin to perform this task.

2.2. Clone all the views into the Default folder. To do this, you need to copy each of the six

provided files into the ELN system:

https://zenodo.org/api/records/13254124/draft/files/Compass.json/content

https://zenodo.org/api/records/13254124/draft/files/Create_Matrix.json/content

https://zenodo.org/api/records/13254124/draft/files/Generate_Matrix.json/content

https://zenodo.org/api/records/13254124/draft/files/Home.json/content

https://zenodo.org/api/records/13254124/draft/files/Multi_Block_PCA.json/content

https://zenodo.org/api/records/13254124/draft/files/OPLS.json/content

2.3. Right-click on the web page and click on “Utils” -> “Copy view” as shown in the screenshot below.

2.4. Open the web page at http://localhost/on-tabs. In the upper-right menu, click on the "Paste View" button and paste the content from the clipboard. Then, click on the "Paste" button to proceed.

2.5. In the upper menu, click "View Manager," type a name for the view, and click on the "Save as" button.

2.6. Repeat this for each view file as shown in previous steps.

2.7. Once the view is saved, you can start using it by dragging and dropping the demo data or your own datasets.

Note: The link on the Home page will redirect you to the Cheminfo installation. You will need to manually adjust the URLs (https://www.cheminfo.org/flavor/metabolomics/) to point to your local installation. For further information, please see <https://docs.c6h6.org/docs/eln/installation/customization>.

**1.6 Short videos/ modules**

A series of short video modules have been created to describe various features within the Metaboscope software.

***Module 1: Landing page and uploading data:***

This module explains the overall architecture of the software suite stratified into three components: data upload, basic data modeling (PCA and OPLS-DA) and advanced modeling (STOCSY, multiblock PCA and COMPASS).

- Select a function from the landing page choosing from 1 (data importation), 2 (PCA and OPLS-DA modeling), or 3 (advanced modeling using multiblock PCA, STOCSY or COMPASS), Figure 1.1. Note -stages 2 and 3 cannot be completed without performing stage 1 first.

**Figure 1.1:** Landing page of Metaboscope.

- Stage 1
- For data importation, choose ‘import from users files’ tab if you want to use your own spectral data set or choose ‘create simulate data’ if you want to create your own spectral dataset using simulated spectra.
- If you prefer to test the platform with the dataset described in the video tutorials as a worked example, use the ‘rodent model of bariatric surgery’ demo data set provided. Click on ‘import from users files’ tab on the home page and select the button ‘Load demo’ in the bottom right-hand corner of the window.
- You will find 3 other available data sets that can be downloaded from the ‘Demo Data’. These datasets are described in the previous section: i) bariatric surgery: rat mode; ii) SARS-CoV-2 infection (a Covid-19 recovery data set); iii) HIMS (the health in men study); iv) INTERMAP Study (an epidemiological study linking diet and cardiovascular risk factors). These demo data sets allow users to experiment with different human biofluids: urine (INTERMAP) and plasma (HIMS and COVID-19 data sets) in order to develop experience with modelling spectral data from human clinical studies.
- Stage 2
- To create a PCA model of your own spectral dataset previously imported and processed in stage 1 or to use the demo datasets, choose ‘Create PCA Model’ in the Stage 2 window on the home page
- To create an oPLS-DA model of your own spectral dataset previously imported and processed in stage 1 or to use the demo datasets, choose ‘Create oPLS-DA Model’ in the Stage 2 window on the home page.
- Stage 3
- To model your own spectral dataset or demo datasets previously imported and processed in stage 1 using multiblock PCA, choose ‘Use Multiblock PCA’ or
- To model your own spectral dataset or demo datasets previously imported and processed in stage 1 using STOCSY, choose ‘Biomarkers identification using STOCSY’
- To model your own spectral dataset or demo datasets previously imported and processed in stage 1 using COMPASS, choose ‘Apply COMPASS’.

***Module 2: Uploading spectral data:***

The user can choose whether to upload data into the web browser using

- their own data and annotations,
- one of the demonstration datasets provided,
- or a simulated dataset based on a list of chemical compounds.

NMR spectral data can be used without transformation. The spectra are linked to an annotation file allowing the assignment of spectra to specific classes, for example, male vs female or disease vs control which must be contained in a separate file. The data files are then exported in a .tsv format prior to performing statistical analysis.

- To import spectra directly from Bruker or JCAMP-DX format, (JEOL format can be implemented on request) drag a zip file from your computer into the drag-and-drop box in the top left panel (Figure 2.1).

**Figure 2.1:** Use the “Drag and drop” box to import the data.

- For each dataset, all the required Bruker folders or JCAMP files should be zipped into a single file, Figure 2.2.

**Figure 2.2:** A single zip file containing NMR spectral for importing the data.

- The loaded spectral data will appear in the panel directly below the drag-and-drop box as single NMR folders or files, as highlighted in Figure 2.3.
- To display ALL the spectra in the list, click on the + icon at the top right-hand side of the panel header, Figure 2.3.

**Figure 2.3:** NMR spectra loaded into the Metaboscope window.

the data.

- Spectra will appear in the bottom centre panel in a superimposed format, Figure 2.4.

**Figure 2.4**: Successfully load NMR spectra are superimposed on the bottom middle panel.

the data.

- Next, navigate to the Excel sheet containing the annotation for the spectra. An example of the excel sheet is shown in Figure 2.5. *Note: the annotation file must contain the experiment number (Exp No) so that it can be matched to the NMR spectrum as below.*

**Figure 2.5**: An example of annotation file containing Exp No.

the data.

- Load an excel sheet containing the annotation for the spectra by dragging the file to the same drag-and-drop box in the upper left corner of the screen as shown in Figure 2.1.
- The metadata are now displayed in the upper right panel of the screen, Figure 2.6.

**Figure 2.6:** Display of metadata in the Metaboscope.

the data.

- To color the spectra according to class (e.g. male versus female or disease versus control), click on the color palate in the header in the top panel, second from the left, Figure 2.7.

**Figure 2.7:** Use the color palate to code the spectra according to class.

the data.

- The spectra will be colored according to the information in the category column. In this example using the rodent bariatric surgery model, the spectra are colored as either pre- (yellow) or postdose (red), Figure 2.8. You can have more than one category column in a spreadsheet, for example, age, sex and disease class.
- In the spectral visualization panel (bottom, center) as highlighted in Figure 2.8, use a left click to place your cursor and then drag and drop to zoom into a spectral region of interest. Double-click to zoom out.

**Figure 2.8:** The spectra are colored as either pre- (yellow) or post dose (red).

- To change the class of interest, for example, to color the spectra by gender instead of pre/post-surgery uncheck the category box in the ‘class’ row and tick the category box in the ‘gender’ row in the list of available metadata in the top right-hand panel (red square). Theb, click on the color palate (blue square) in the header in the top panel, Figure 2.9.
- To download the dataset, use the download key (orange square), Figure 2.9.

**Figure 2.9:** Change the color coding in the spectra from class to gender.

***Module 3: Data processing***

The data processing module enables the user to pre-process the spectral dataset using functions such as the exclusion of unwanted spectral regions (for example the residual water resonance or drug metabolites), changing the spectral resolution, or performing different normalization algorithms.

- To import a data file, drag and drop the data file (saved from the import function in video 2 or from the demo datasets such as Bariatric, Covid, HIMS and INTERMAP) from your computer into the drag-and-drop box in the top left panel of the screen. Alternatively, click on the demo data tab in the bottom right-hand side of the screen and choose the bariatric dataset.
- The spectra will appear in the ‘list of spectra’ panel on the left-hand side of the screen just underneath the drag and drop box and the corresponding annotations will appear in the top right panel, Figure 3.1.

**Figure 3.1**: Successfully loaded spectra and the annotation file are shown in the highlighted section of the page.

- In order to color spectra according to class, check the ‘class’ box under the ‘category’ column in the list of annotation data (red box, Figure 3.2).
- Click on the ‘+’ icon in the ‘list of spectra’ to display all the spectra, Figure 3.2.

**Figure 3.2**: Use the ‘+’ sign to display the spectra data

- Click on the color palate and the spectra will be color-coded according to class. In the case of the bariatric surgery rat model, this will give 4 classes: baseline and 2, 6, and 8 weeks following surgery, Figure 3.3.

**Figure 3.3**: The bariatric dataset is color-coded according to the timepoint pre or post surgery.

- Explore the spectra by zooming in and out of regions of interest by left-clicking to place the cursor and dragging over the region of interest, using the middle bottom panel.
- Scroll up with the mouse to expand vertically.
- Double-click to return the spectra to their original dimensions.
- To exclude spectral regions that are not interesting or that will add noise to your models use the ‘preferences to generate the data’ panel to define regions that you want to exclude. For example, because the regions below 0.5 ppm and above 9.8 ppm contain only noise (with the exception of TSP at 0.0 ppm, which should typically be removed prior to modeling the data), set the range from 0.5 to 9.8 ppm as shown and click ‘reset’, Figure 3.4.
- The spectral region containing the residual water resonance and the urea peak (which is affected by cross saturation of protons from water) can be removed using the ‘exclusion’ function in the ‘preferences to generate the data’ panel. To remove further regions, click on the + sign next to the ‘Exclusions’ icon and insert a second ppm range. A suggested exclusion range would be from 4.7 to 4.9 ppm (water) and from 5.5 to 5.9 ppm (urea), Figure 3.4
- To reinsert an excluded region, click on the ‘-‘ sign next to the relevant ‘Exclusions’ icon
- To increase or decrease the spectral resolution, click on the ‘No. points’ icon
- To scale the spectral data, go to ‘Matrix processing’. The following scaling functions are provided in a drop-down menu: probabilistic quotient normalization (PQN); mean center data; scale to the tallest peak. Click on the scaling function of your choice.
- To remove the scaling factor, click on the ‘-‘ sign.

**Figure 3.4:** Change the range of the spectra region to 0.5 to 9.8ppm.

- Change the display drop-down menu in the bottom center panel to normalized, Figure 3.5

**Figure 3.5**: Change the display drop-down menu to “normalized” to display the normalized data.

- Download the processed data by clicking on the downward arrow, Figure 3.6. The exported file can be found in your download folder.

**Figure 3.6**: Use download tab to download the processed data.

***Module 4: Principal components analysis (PCA)***

The module allows the user to perform PCA on the pre-processed datasets interactively. The user can interrogate the model scores and loadings and remove ‘outliers’ from the model where desired. The PCA model is automatically recalculated on the removal of outliers.

- Click on the ‘Model your own PCA data’ from Stage 2 of the landing tab. Drag and drop the processed .tsv file that you prepared in video 3 into the top left-hand panel, Figure 4.1

**Figure 4.1**: PCA page.

- The list of spectra and the processed spectral data are shown in the bottom right and bottom center panels accordingly, Figure 4.2.

**Figure 4.2:** Display of processed spectral data.

- Spectra are color-coded according to the class. The spectra can be viewed interactively by zooming into spectral regions of interest (click and drag the cursor to define the region, double click to return to the full spectrum). Refer to video 2 if you would like to change the category from class to another metadata parameter e.g. sex
- To calculate a PCA model, click on the ‘calculate/recalculate PCA’ tab and the PCA scores plot will be displayed in the top center panel, color-coded according to class. The number of samples in the dataset and the number of samples used to calculate the PCA model is also displayed (see red rectangle, Figure 4.3).

**Figure 4.3:** Display of number of spectra used for PCA calculation.

- The scores plot is displayed once calculation has been completed, Figure 4.4.

**Figure 4.4**: Display of PCA scores plot.

- To show the category box, check the ‘show category’ box, and to show the sample ID check the ‘Show ID’ box in the PCA panel header, Figure 4.5.

**Figure 4.5:** Use the category and show ID checkbox to display the information on the scores plot.

- To change the principal components plotted, click on the drop-down menu for the X and Y axes at the left-hand side of the header and select from the menu, as highlighted in Figure 4.6. *Note different combinations of PCs will uncover different inherent patterns in your dataset. For more information on PCA please see (Jolliffe and Cadima, 2016).*
- The 95% confidence limit or Hotelling’s T-test boundary is shown by the ellipse, colored by class. This can be switched on and off using the ‘Display ellipses’ check box at the top of the PCA scores plot, red box in Figure 4.6.

**Figure 4.6:** Use the drop-down menu to change the principal components to be plotted and use the check box to display the Hotelling’s T-test boundary.

- To see the different options for viewing the PCA scores and loadings, click on the ‘PCA-PC’ tab at the top left of the screen, Figure 4.7.

**Figure 4.7:** Use the PCA-PC tab to view different PCA scores and loading plots.

- This window provides the user with different paired combinations of PC scores on the left-hand side of the screen and the corresponding loadings on the top right-hand side of the screen. The explained cumulative variance is provided in the bottom right-hand side of the screen, Figure 4.8

**Figure 4.8**: Quick glance of the pairwise comparison of the PCA scores plot together with the loadings for each component.

- Visualization of the loadings is interactive and as with the spectra, you can zoom in and out of spectral regions by left-clicking and dragging the cursor over the region of interest. To expand/contract vertically scroll up/down with the mouse, Figure 4.9.

**Figure 4.9:** Predictive and orthogonal components visualization panel can be viewed by zoom in and out.

- Use the PCA tab to return to the main PCA model menu.
- If you want to build a PCA model with a subset of the data or to explore different two-class combinations, select the spectra you want to include in the model from the ‘List of spectra in dataset’ menu at the bottom right-hand side of the screen.
- Samples with a green tick in the column headed by the ‘TICK’ in the ‘list of spectra in the dataset’ are displayed in the spectral visualization panel. Samples with a green tick in the column headed by ‘PCA’ are used to build the PCA model (red box, Figure 4.10).

**Figure 4.10:** Information display in the list of spectra in dataset panel.

- To select specific samples, use the shift button on your keyboard and hold down to highlight the first sample you want to select. Move the cursor down to continue selecting samples until you have finished the selection.
- To deselect samples, highlight and click on the ‘-‘ sign in the ‘List of spectra in dataset’ header. To reinsert samples that have been excluded, highlight the samples and then click the ‘+’ icon in the header (red circles, Figure 4.10).
- Only the selected samples will appear in the spectral visualization panel once the drop-down ‘display’ menu is selected, Figure 4.11.

**Figure 4.11:** Display of selected spectra using the drop-down menu option.

- Samples that have been excluded will still appear in the PCA scores plot in the principal component analysis window but will appear as smaller circles, Figure 4.12. *Note: at this stage, the PCA model has not been recalculated and only the visualization properties have been changed.*

**Figure 4.12:** Display of scores plot based on the selected spectra.

- To recalculate the PCA model with the newly selected samples, click on the ‘calculate/recalculate tab at the top of the screen, Figure 4.13. Only the selected samples have now been used to calculate the PCA model and all other samples in the spectral dataset are projected onto the model as open circles.

**Figure 4.13:** Recalculate PCA and the display scores plot included the projection of the excluded samples in open circles.

***Module 5: Features for visualization***

This module covers the use of the features in the visualization panels. Statistical TOtal Correlation SpectroscopY (STOCSY) (Cloarec, et al., 2005) is described in this module to aid the identification of signals of interest identified in the loadings plots. STOCSY calculates the Spearman’s correlation between each data point in a spectrum to derive a correlation matrix for a given set of spectra. STOCSY uses the correlation of the intensity variables in a spectrum and identifies the presence of multiple peaks from the same compound in a set of NMR spectra. An additional feature in the data visualization panels is the ability to rank data point intensities to establish which metabolites are most abundant for a given metabolite or signal.

- Load the .tsv file containing the processed data from Module 3 or Module 4 and perform PCA (see Module 4 for instructions). In order to sort samples according to the category, click on the ‘Category’ column heading in the ‘List of spectra in dataset’.
- As for Module 4, exclude the samples you do not want to be included in the model first. Perform this by selecting spectra by holding down the shift key and scrolling down.
- Remove the spectra from the visualization panel display but clicking on the ‘-‘ icon in the header. Repeat the procedure to remove further samples.
- To bring back a sample that you have previously excluded, select the sample and click on the ‘+’ icon in the header.
- In order to help identify correlated signals, you can perform STOCSY.
- Zoom into a peak of interest in the spectral visualization panel. Hold down the SHIFT and ALT keys simultaneously and click on the apex of the peak of interest, Figure 5.1.

**Figure 5.1**: Use the Shift+ALT key to click on the apex of the peak of interest to obtain the STOCSY results.

- All data points in the spectra will now be color-coded according to their correlation with your selected peak. Thus, the highest correlations (coded in red) will generally be those adjacent to the selected data point as they are part of the same spectral peak. All related peaks will be identified by red or orange coloring signifying a strong correlation, Figure 5.2.

**Figure 5.2**: The STOCSY output for the apex of 7.57ppm.

- The initial selected data point corresponding to the apex of your peak of interest is highlighted on the spectrum by a vertical yellow bar, Figure 5.3. The expansion of the aromatic spectral region shows the correlation for the signals corresponding to the aromatic ring protons for hippuric acid, Figure 5.3.

**Figure 5.3:** Expansion of the STOCSY with the vertical yellow bar corresponding to the apex of the peak of interest.

- To remove the correlation color line plot from the display, select ‘none’ from the drop-down menu in the spectral visualization window, Figure 5.4. To display the selected spectra, use the drop-down ‘Display’ menu and check ‘selected’, or to redisplay the whole spectral dataset select ‘All’.

**Figure 5.4:** Use the correlation drop-down menu to change the display of the visualization plot.

- Metabolites can be ranked by their intensity, reflecting their concentration. Tick the ‘tracking info’ box on the header of the spectral visualization panel. Spectra will be ranked in descending order based on the cursor position (X value), Figure 5.5.

**Figure 5.5:** Tick the tracking info box on the header of the visualization to display peak height ranked in the descending order.

- The scores plot and the sample list are linked. In order to identify specific samples, hover the cursor over a chosen coordinate in the scores plot, and its identity will be shown in the sample list, Figure 5.6.
- To exclude this sample from the analysis, select the highlighted sample in the ‘List of spectra in the dataset’ and click on the ‘-‘ sign (red circle, Figure 5.6) The excluded sample will be shown as a smaller dot in the scores plot. *Note: the PCA model has not been recalculated in Figure 5.6.*

**Figure 5.6:** The sample list and scores plot is interactively linked.

- To explore the scores plot, left-click and drag the cursor over the area you want to expand. Double-click to zoom out.
- To recalculate the PCA model without the excluded sample, click on the ‘Calculate /recalculate’ tab at the top of the screen. The excluded samples are not used to build the model but are projected onto the model as open circles.
- To select the number of standard deviations to display in the scores plot select the required number from the drop-down ‘No. SD’ menu, Figure 5.7.

**Figure 5.7:** Use the No. SD drop-down menu to change the number of standard deviations to be displayed on the scores plot. In this example, we have chosen 3.

***Module 6: Features in the visualization II: Box plot***

This module is an extension to module 5 that covers additional features that are available in the visualization panel. Box plot statistic is implemented in this module and provides information on minimum, maximum, and quartile values. The ability to simultaneously display both the spectral data and statistics on the visualization panel enables the user to make comparisons of the data easily.

- Load the .tsv file containing the processed data (see Module 3 or 4).
- Select or remove relevant spectra in the dataset by clicking on the ‘+’ or ‘-‘ icon in the List of spectra in the dataset (see Module 4).
- The visualization panel (green box, Figure 6.1) allows you to indicate what information is to be displayed. To display the spectra, use the Display drop-down menu (red box. Figure 6.1). Change the drop-down menu to “all”, to display all spectral data in the dataset, choose “selected” to display spectra that have been selected or “none” if you do not want to display any spectral data in the visualization panel.

**Figure 6.1:** PCA page with the visualization panel.

- The same visualization panel can also be used to display the box plot statistics. Similar to the spectral data display, the user can choose to display the box-plot statistics for “selected”, “all” or “none” of the spectral data using the drop-down menu in Box-plot (red oval, Figure 6.2).

**Figure 6.2:** Use the box-plot drop-down menu to select the statistics for the corresponding dataset.

- The box-plot statistics are displayed in the following format (Figure 6.3): pale grey shading indicates the range between minimum and maximum values for each data point, and dark grey shading indicates the range between the first and third quantile whilst the median value is indicated using a solid line.

**Figure 6.3:** Visualization panel showing box plot statistics.

- To display both the spectral data and box-plot statistics in the visualization panel, choose the appropriate combinations using both the “Display” and “Box-plot” drop-down menus (red box, Figure 6.4). The figure below shows three representative spectra which have been overlayed with the overall dataset statistics. This allows trends in the data to be compared.

**Figure 6.4:** Use the Display and Box-plot drop-down menus to choose what information to be displayed on the visualization panel.

***Module 7: Orthogonal Projection to Latent Structure Discriminant Analysis (OPLS-DA)***

In this module, the calculation of OPLS-DA models is explained (Bylesjo, et al., 2006). This tool is used to maximize separation between two or more classes and to map test sets onto the calculated OPLS-DA model to predict class membership. The output panels are used to explore the contributions of metabolites to the predictive component and to the orthogonal component. The code was implemented in accordance with metabom8 package available at https://github.com/tkimhofer/metabom8.

- Click on the ‘Model your own OPLS-DA data’ from the landing tab. Drag and drop the processed .tsv file that you prepared in module 3 into the top left-hand panel (red box in Figure 7.1). The list of spectra and the processed spectral data are shown in the bottom right (purple box) and bottom center (brown box) panels accordingly.

**Figure 7.1**: OPLS page showing the data importation panel (red box), visualization panel (brown box), and the list of spectra in the dataset (purple box).

- To utilize various features in the visualization panel, refer to Modules 5 and 6. Refer to Module 2 if you would like to change the category from class to another metadata parameter e.g. sex.
- If you want to build an OPLS-DA model with a subset of the data, select the spectra you want to include in the model from the ‘List of spectra in dataset’ menu at the bottom right-hand side of the screen. Select or remove relevant spectra in the dataset by clicking on the ‘+’ or ‘-‘ icons in the List of spectra in the dataset (see Module 4).
- To calculate an OPLS-DA model, click on the ‘calculate/recalculate OPLS’ tab (yellow box, Figure 7.2) and the OPLS scores plot will be displayed in the top center panel (green box), color-coded according to class. The number of samples in the dataset and the number of samples used to calculate the OPLS-DA model are indicated in the top right-hand corner (blue box). The visualization features in the OPLS-DA scores plot are the same as in the PCA module.
- The scores plot (green box, Figure 7.2) is interactively linked to the sample list (purple box). Use the cursor to hover over the data point on the scores plot to see the identity of the sample as highlighted in the sample list.
- The projection of the predicted scores onto the OPLS-DA model (cyan box, Figure 7.2). Samples used to build the OPLS-DA are shown in closed circles whilst samples that have been excluded are shown in open circles. Samples are color-coded according to the time category (time before/since surgery).

**Figure 7.2:** OPLS page showing a two classes OPLS-DA model and the data selected for the model.

- To see the seven-fold cross-validated scores, click on the ‘OPLS-CV tab at the top left of the page (yellow box, Figure 7.3).

**Figure 7.3:** OPLS page.

- The cross-validated scores plots are shown on the top left panel (green box, Figure 7.4). The coefficient plot for the predictive component is shown in the middle top panel whilst the orthogonal component is shown in the top right-hand panel. The predictive component is related to the differentiation between the two classes in the OPLS-DA model, whilst the orthogonal component shows systematic variation related to the dataset but are not related to the two classes. These plots are interactive so the user can click and drag, or scroll up and down, using the mouse to expand horizontally or vertically, respectively.
- The OPLS-DA model statistics are shown in the bottom panel (red box, Figure 7.4). The red bar in the bar plot shows Q^2^Y, the green bar indicates the area under (AUC) the ROC curve, blue for R^2^Y, and grey for R^2^X. The Q^2^Y provides information on the predictivity of the OPLS-DA model. R^2^Y explains the variation in the total dataset relating to the class and R^2^X describes the variation in the spectral data explained by the OPLS-DA model. A high Q^2^Y and AUC are desirable in an OPLS-DA model.

**Figure 7.4**: Display of the OPLS cross-validated scores (green box), predictive and orthogonal components as well as the model statistics (red box).

- To return to the scores plot of the OPLS-DA, click on the “OPLS” tab at the top left of the page (yellow box, Figure 7.5). To calculate another OPLS-DA model, repeat the procedure described here.

**Figure 7.5**: Use the OPLS tab to return to the OPLS scores plot.

***Module 8: Multiblock PCA***

To overcome issues arising from dynamic range problems, where high-concentration metabolites can dominate the model over lower-concentration, but biologically relevant metabolites, multiblock analysis can be performed in conjunction with either PCS or OPLS-DA. This module allows the user to model selected subsections of the spectrum with options to scale or pre-process each block individually. Scores and loadings or coefficient plots can be displayed individually to provide finer detail of the biologically relevant structure in the dataset (Loo, et al., 2021).

- Click on the ‘Use the Multiblock PCA” tab from the landing tab. Refer to previous modules on how to utilize various features in the visualization panel, as well as how to perform STOCSY. To perform multi-block PCA, drag and drop the processed .tsv file that you prepared into the top left-hand panel, Figure 8.1.

**Figure 8.1:** Drag and drop the processed data file into the top left-hand panel.

- By default, spectra are divided into equal regions of 0.5 ppm (Figure 8.2). To calculate multi-block PCA, click on the “calculate/recalculate PCA” tab (in blue).

**Figure 8.2**: Spectra are divided into equal regions of 0.5 ppm for multi-block PCA.

- Once the multiblock PCA model has been calculated, each individual PCA sub-model corresponding to the sequential 0.5ppm is listed in the calculated blocks in the bottom left panel (green box, Figure 8.3), each labeled by the block range in ppm. Click on the block or spectral region of interest in the list to display the PCA scores plot for that region in the visualization panel. In Figure 8.3, block b2 for the ppm range 0.5 to 1.0 ppm is selected. The spectral data for the selected block are displayed directly above the scores plot. The user can zoom in and out of these visualization plots interactively as shown in previous modules.
- To review the scores and loadings plots easily. Click on the “PCA PC” tab on the top left-hand side (yellow box, Figure 8.3).

**Figure 8.3**: Multiblock PCA showing scores plot and corresponding spectral data for the block between 0.5 to 1.0 ppm region.

- Use the “PCA PC” tab (highlighted in the green box, Figure 8.4) to navigate to the page that gives a quick summary of the contribution of each principal component for the selected PCA block. The left-hand panel shows the list of calculated blocks, the middle panel shows the principal components plotted in a pairwise fashion and the right-hand panel shows a stack of four sub-panels. (Figure 8.4). The top two panels are derived from the PCA model, calculated using full spectral region. The first sub-panel from the top is the average spectrum color-coded according to the standard deviation and the second sub-panel is the loading plot. The bottom two sub-panels show the same information but are calculated using only the selected region of the multi-block PCA model, in this case, the block for 1.5 to 2.0 ppm (Figure 8.4). Having the plots of the standard PCA and multi-block PCA on the same page enables the user to compare the effect of the standard versus the multiblock approach on the model loadings easily.
- On the “PCA PC” page, the user can change the component for the PCA model corresponding to a single block using the “MBPCA component” drop-down menu. To change the component for the PCA model of the full spectral range use the “PCA component” drop-down menu instead (orange box, Figure 8.4). In many cases, the multi-block approach allows the user to see more subtle effects compared to using the global model. The user can zoom in and out of these four sub-panels.

**Figure 8.4**: The PCA PC tab on the multi-block PCA module (orange box) gives a quick summary of the loading and scores plots for the selected block, in this case, the block between 1.5 to 2.0 ppm is selected the block.

- To display the processed spectra and the cumulative variance explained by the model, click on the 'Compare Loadings' tab next to the “PCA PC” tab, purple box in Figure 8.5. The top left panel contains the processed spectra, and the top right shows the cumulative variance explained by adding sequential components to the model. The loadings and standard deviation spectrum for the full spectrum (two sub-panels in the middle) and the selected multi-block (bottom two sub-panels) in the PCA-PC page are also displayed below on this page. Users can choose which block of information to be displayed using the list on the left-hand side, as well as zoom in and out of these panels.

**Figure 8.5**: The “compare loadings” page within the multi-block PCA module.

- To directly compare information from the loadings associated with each PC, use the “Loadings” tab, Figure 8.6. The heading on each panel describes the content (orange boxes, Figure 8.6).

**Figure 8.6**: The loadings page within the multi-block PCA module.

- The user can change the width of the blocks or create blocks of unequal size. To do this, click on the “home” tab. Then, tick the "selected blocks" option, in the configuration panel (orange box, Figure 8.7).

**Figure 8.7**: The user can set different block sizes using the options available in the configuration panel.

- The user can define the range for each block and assign a label. These regions will be denoted in the spectral visualization panel, by a solid red bar at the top of the spectrum. Regions do not have to be consecutive. In this example, the defined block is from 2.3 to 2.8 ppm, Figure 8.8. To add the next block, just repeat the same process. Users can define as many blocks as they want. For this illustration, we have defined two specific regions of interest.

**Figure 8.8**: Two non-equal block sizes have been set using the list of ranges panel.

- The user can click on the “calculate/recalculate PCA” tab on the home page to perform multi-block PCA. Alternatively, the user can fill the gaps between these selected blocks using the "fill the other block" check box in the configuration panel, Figure 8.9.

**Figure 8.9**: The configuration panel block options include filling the other blocks which have not been defined by the user.

- Use the “fill the other blocks” option, the user will now have 5 independent blocks, Figure 8.10. Blocks that have been defined by the users are indicated by a red solid bar whilst those that fill using the “fill the other blocks” option are indicated using a blue box.

**Figure 8.10**: The visualization panel shows 5 independent blocks have now been defined.

- To generate multi-block PCA of these 5 blocks, click on the "calculate/recalculate PCA" tab on the home page. Figure 8.11 shows multi-block PCA models based on these 5 non-equal block sizes.

**Figure 8.11**: Multi-block PCA on 5 independent blocks with unequal block size.

***Module 9: COmbined Multi-block Principal component Analysis with Statistical Spectroscopy (COMPASS)***

The COMPASS (Loo, et al., 2021) module provides an easy way of searching for specific spectral patterns in a dataset, in order to identify how many samples in that dataset contain a particular metabolite. For example, it may be relevant to know how many participants have taken a particular medication such as ibuprofen. The user defines a ‘reference’ pattern based on a spectrum where the signal pattern for ibuprofen or a related metabolite is clear, not overlapped, and present in relatively high intensity. The selection of the ‘reference’ pattern can be achieved by ranking the available samples in descending order of intensity, allowing the user to select the sample with the highest concentrations of that metabolite. A threshold can be defined in order to allow the unambiguous identification of samples containing the reference pattern. Samples containing the reference compound matches are ranked in descending order of intensity, allowing the user to select the sample with the highest concentration of that metabolite for further structural elucidation, if necessary.

The module allows the user to perform COMPASS on the pre-processed datasets interactively. COMPASS involves choosing a reference pattern to identify how many samples in the dataset share the same pattern. The reference pattern is usually related to a specific chemical compound and contains one or more signals in the spectral pattern. An ideal reference pattern is where the pattern in the spectrum is clear, high in intensity, and shows minimal overlap with signals from other molecules. This optimal reference pattern that has been chosen as the “target reference pattern” by the user is then used to calculate the cross-correlation (CC) for the same spectral area in each spectrum in the dataset. The distribution of the CC for all samples in the dataset is displayed. A CC threshold can be defined by the user to determine the likelihood that the reference pattern is present in a particular spectrum. This process can be used to provide a rapid estimation of the number of samples containing the compound represented by the reference pattern.

- Click on the ‘Apply COMPASS’ tab from the landing page.
- To define the ppm range for a selected peak in the spectrum, use the ALT key on the keyboard and click at the beginning and the end of the ppm range using the cursor (brown box, Figure 9.1). The selected ppm range is highlighted in the visualization panel with a red bar above the selected signal and listed under the “List of ranges” panel (blue boxes, 9.1).

**Figure 9.1**: Selection of a particular cluster of peaks for COMPASS.

- For a metabolite of interest that contains multiple peaks in the spectrum, the user can define multiple ppm ranges using the same procedure. To identify multiple signals that are derived from the same molecule, the user can use STOCSY (refer to Module 5). In general, the greater the number of signals that have been defined in the reference spectrum, the more accurate the pattern matching will be.
- To identify a spectrum with high peak intensity, tick the “tracking info” checkbox (cyan box, Figure 9.2). This will display the sample ID in the visualization panel in descending order based on a specific data point pinpointed by using the cursor. In Figure 9.2, the selected reference region is between 2.29 to 2.31ppm as defined by the user and listed in the “list of ranges” (blue box). The user can zoom in on the peak of interest in the visualization panel and use the cursor to pinpoint the apex of the peak. In this case at 2.30147 ppm. As the “tracking info” checkbox has been checked (cyan box, Figure 9.2), the spectra IDs are ranked in descending order. It can be noted that sample ID BariatricRats/428 has the highest peak intensity. As mentioned before, an optimal reference spectrum should contain a high peak intensity of the peak of interest, and the peak should have minimal overlap with other peaks.

**Figure 9.2:** Use the tracking info to rank the spectra peak intensity in descending order.

- To set the peaks in the spectrum from sample ID 428 as the target reference pattern, navigate to the “List of spectra in dataset” panel and click the set target symbol (Figure 9.3). The spectrum that has been set as a target reference pattern is highlighted in green in the “list of spectra in dataset” panel (Figure 9.4).

**Figure 9.3:** Setting the target reference spectrum using the set target symbol in the “list of spectra in dataset” panel.

**Figure 9.4:** Spectrum ID 428 has been set as a target reference spectrum highlighted in green in the “List of spectra in dataset” panel.

- Select the peak of interest in the “List of ranges” panel (blue box, Figure 9.5) to obtain the CC values for each of the spectra in the dataset and relative to the target reference sample (i.e. spectrum ID 428). In Figure 9.5, we selected peak 2.30 ppm (ranges between 2.29 to 2.31 ppm). The scatter plot of the CC values for each spectrum is displayed on the top right-hand panel as highlighted. The scatter plot is color-coded according to the time category of the samples.

**Figure 9.5:** Scatter plot of the cross-correlation output is displayed in the top right-hand corner and the samples are color-coded according to the time category.

- To set the threshold of the CC value, use the “Preference” panel. The first dashed line in the scatter plot of the CC visualization panel corresponds to the default threshold value of 0.98. Samples above this threshold line will have a high CC with the selected target reference sample (red arrow, Figure 9.6).

**Figure 9.6:** The use of the “preferences” panel to set the threshold of the CC value in the scatter plot for CC values for each sample.

- The second dashed line in the scatter plot of the CC visualization panel corresponds to the second threshold in the “preferences” panel which is by default set at 0.8. Samples between the first and second thresholds will have a slightly lower CC value than the target reference sample (red arrow, Figure 9.7) compared to those above the first threshold. This indicates that the samples between these two thresholds are likely, but not certain, to contain the peak of interest.

**Figure 9.7:** The use of the “preferences” panel to set the threshold of the CC value in the scatter plot for CC values for each sample.

- In the bottom right-hand visualization panel, change the drop-down menu of “Correlation” to COMPASS (yellow box, Figure 9.8). Then, check the “Data to display COMPASS” in the “Preferences” panel to “Values > the Second threshold”. In doing so, this will display all the spectra above the second CC threshold at 0.8 in the visualization panel.

**Figure 9.8:** Change the correlation drop-down menu to COMPASS (yellow box) in the visualization panel and the data to display COMPASS to values > second threshold in the “Preferences” panel to display spectra that have a CC value above 0.8 in the visualization panel.

- The user can zoom in on the region of interest to inspect the spectra. Since the spectra are color-coded according to the class (time category in this case), this allows the user to check major class differences in the intensity of the peak visually. In Figure 9.9, we can see that baseline samples (color-coded to red) do not have the peak of interest. Moreover, the CC scatter plot in the top right-hand panel also shows that the baseline samples tend to have lower CC values than samples at weeks 2, 6, and 8.

**Figure 9.9:** Zoom in on the region of interest to inspect spectra at a glance for those samples with CC greater than the second threshold of CC value at 0.8.

- As the process is interactive, this enables the user to adjust the second threshold level based on this information. Change the second threshold to 0.88 (purple box) and zoom in to the same region again to visually check all samples with CC > 0.88 (Figure 9.10).

**Figure 9.10:** Use the “Preferences” panel to increase the second threshold to 0.88 and repeat the visual inspection on the 2.30ppm region.

- Similarly, the user can adjust the first threshold depending on how confident the user is that the reference pattern is present for a given CC value in the sample. Figure 9.11 shows the threshold has been changed to 0.95 and the “Values > First threshold” has been checked for the “data to display COMPASS” (purple box). The visualization panel in the bottom right-hand panel now shows spectra with CC values > 0.95.

**Figure 9.11:** Use the “Preferences” panel to reduce the first threshold to 0.95 to display spectra with CC >0.95.

***Module 10: COMPASS and STOCSY***

This module describes how to combine the functions of COMPASS and STOCSY in order to improve the certainty of capturing the true molecular signature of any given metabolite. By modification of the correlation threshold for STOCSY, it is possible to focus on a single molecule, or often to identify other molecules in shared pathways or arising from shared biological functions, which can often be achieved by lowering the correlation threshold.

- Click on the ‘Apply COMPASS’ tab from the landing page. In , we use sample ID 428 as the target reference sample since it contains the highest peak intensity for the peak of interest at 2.30 ppm. The threshold for CC values was set at 0.95 and 0.8. To enable the user to select a subset of data with CC > 0.95 for STOCSY, we only display the spectra with CC values greater than 0.95 on the visualization panel. Refer to Module 9 on how to set the target reference, select the reference region, then set the CC threshold, and display selected spectra on the visualization panel.
- To perform STOCSY on the subset of data with CC > 0.95, we use the apex of the peak at 2.30 ppm, as the driver, by using the shift and ALT keys on the keyboard to allow the user to define the apex of the peak of interest. Refer to Module 5 on how to perform STOCSY.
- To display the STOCSY plot without the spectral data, change the display drop-down menu to “none” (red box, Figure 10.1). Peaks with high correlations to peak 2.30 ppm are shown in a warmer color, on the visualization panel on the right-hand bottom panel.

**Figure 10.1**: Display of the STOCSY plot based on the samples with high CC values for the peak at 2.30 ppm.

- Zoom in to the peaks with warmer colors around 6.8 to 7.9 ppm. You can see that peaks at 7.05 ppm and 7.22 ppm, are colored red. This indicates that these peaks are most likely derived from the same molecule as the driver peak at 2.30 ppm, Figure 10.2. Indeed, these three peaks correspond to the p-cresol sulfate molecule, which is a gut microbial metabolite.

**Figure 10.2**: STOCSY plot that shows peaks at 7.05 and 7.22 ppm are highly correlated to 2.30 ppm.

- To add these extra signals to our reference pattern, we now choose “selected” in the display drop-down menu (orange box, Figure 10.3) in the visualization panel to visualise the relevant spectral data. Zoom in to the peak at 7.22 ppm and define the ppm range for COMPASS. As before, a red line above the peak range, now labelled as peak B, can be seen on the visualization panel. The “List of ranges” panel also shows both selected regions (blue box, Figure 10.3).
- To create a reference pattern that includes both peaks at 2.30ppm and 7.22 ppm, select Range A, and Range B, from the list in the ranges panel, and click on the “Join ranges” tab (red box, Figure 10.3).

**Figure 10.3**: Display of spectral data on the visualization panel with two peaks of interest selected.

- A new range, including both A and B, is created. This is defined as range BA in the “List of ranges” panel (blue box, Figure 10.4). You can now compare the effect of applying the different ranges you have created on the distribution of CC values, in the scatter plot panel. The distribution of the cross-correlation values (red box) is rather different for each pattern listed in the “List of ranges”.

**Figure 10.4**: Scatter plot of cross-correlation value for the reference pattern of 2.30 and 9.22 ppm.

- To establish which samples contain the metabolite of interest, change the “Data to display COMPASS” in the preferences panel, to "values > second threshold" (orange box, Figure 10.5). Ensuring the display drop-down menu, in the visualization panel is set to “selected” (blue box), we can zoom in and have a look at the relevant peaks with CC > 0.8.

**Figure 10.5**: Display of spectra with CC > 0.8 for 7.22ppm.

- Continue to use the combined range but change the second threshold to 0.6. As you can see, despite using a much lower cut-off point of 0.6, all selected samples contain a peak at 7.22 ppm (Figure 10.6). The same is also true for the peak at 2.30 ppm (Figure 10.7). If you remember, in Module 9, we had to set the cut-off at 0.88 for this peak to prevent any false positive results.

**Figure 10.6**: Display of spectra with CC > 0.6 for 7.22ppm.

**Figure 10.7**: Display of spectra with CC > 0.6 for 2.30 ppm

- The next step is to perform STOCSY on 2.30 ppm using the refined subset of spectral data with CC > 0.6 (rather than based on a subset of spectral data with CC >0.88 as shown in Module 9). To perform STOCSY on this subset of spectral with CC > 0.6, change the display to "none", and the correlation to COMPASS. This shows the STOCSY correlation plot, for the driver peak at 2.30 ppm (Figure 10.8). It can be seen that 4-cresol sulfate peaks at 7.05 ppm and 7.22 ppm have a higher correlation to the driver peak at 2.30 ppm than the scenario in Module 9, where the single peak, at 2.30 ppm was used, to define the cut-off values. The STOCSY plot also shows peaks at 7.38 ppm and 7.42 ppm, which are also correlated with the driver peak at 2.30 ppm, but at a lower value than the 4-cresol sulfate peaks at 7.05 ppm and 7.22 ppm. In this case, the lower correlations for peaks at 7.38 ppm and 7.42 ppm indicate a correlation of the 4-cresol sulfate with phenylacetylglycine, which is also a gut bacterial metabolite deriving from the aromatic amino acid phenylalanine.

**Figure 10.8**: STOCSY correlation plot based on spectral data with CC >0.6 shows a higher correlation between the signal at 2.30 ppm and the peaks relating to 4-cresol sulfate at 7.05 ppm and 7.22 ppm but a lower correlation from phenylacetylglycine at 7.38 ppm and 7.42 ppm.

***Module 11: Generate a simulated dataset***

Simulated datasets can be useful for evaluating how class size and number, or the difference in concentrations of biomarkers between classes, affects various modeling functions in the data. Simulated spectra are created using a list of chemicals defined by either SMILES codes or drawn in a chemical structure drawing panel. Once imported the compounds can be named as required.

Biofluid spectra can be simulated by selecting a range of molecules from the list of chemical compounds and the following parameters can be adjusted: the number of classes; identified ‘biomarkers’; the number of spectra per class, and compound concentration ranges. Having created a simulated spectral dataset, all the normal functions of the toolbox (PCA, OPLS-DA, STOCSY, COMPASS, etc) can be used.

- Click on the ‘create simulated data” tab from the landing tab.
- To generate a simulated dataset, drag and drop an SDF file to the bottom left-hand box, which states “drop your SDF file here” (purple box, Figure 11.1). Alternatively, users can create an SDF file by clicking on the “create’ tab on the top left-hand corner (yellow box, Figure 11.1)

**Figure 11.1:** The simulated data page allows the user to generate a simulated dataset using create tab or an SDF file.

- The top left-hand panel allows the user to add the SMILES ID for each compound. Figure 11.2 (blue box) shows an example of 7 compounds that have been added to the panel.
- Click on the “add list of SMILES” button (red circle, Figure 11.2).

**Figure 11.2:** The simulated data page allows the user to generate the data using create tab or an SDF file.

- The list of compounds will appear in the bottom right-hand panel as highlighted in Figure 11.3. To change the column heading, use the parameters panel, on the top right (orange box, Figure 11.3). To add or delete a column, use the plus or minus sign (red circle).

**Figure 11.3:** List of compounds generated using the SMILES list.

- To add the name of each compound, type the information under the column name Figure 11.4

**Figure 11.4:** Add the name of each compound under the column heading.

- You can also draw your own molecule using the drawing interface panel and add it to the list. In Figure 11.5, I have drawn a benzene ring.
- Click on the “Add structure” tab as highlighted in Figure 11.5. The benzene ring will be added to the list of compounds in the bottom right-hand panel.
- To delete any unwanted compound from the list, use the bin icon as indicated in a red circle, Figure 11.5.
- Use the list that you have created, and download it as an SDF file using the download icon as indicated in the blue circle, Figure 11.5.
- This SDF file may be used to create a simulated dataset by returning to the home page using the “home” tab, yellow box, Figure 11.5.
- Drop the SDF file that you have created into the bottom left-hand box which states “drop your SDF file here” (purple box, Figure 11.1).

**Figure 11.5:** Draw your own molecule to add to the compound list, and use the bin icon to remove unwanted compound.

- Alternatively, you can directly import the list of compounds that you have created using the import icon as highlighted in Figure 11.6) on the “Select molecules to be part of the dataset” panel. The compounds list that you have created is also listed in this panel.

**Figure 11.6:** The compound list is loaded on the create simulated dataset page.

- Now, you can select the individual compounds that you want to add to create a spectrum by using the plus sign on the “Select molecules to be part of the dataset” panel (highlight, Figure 11.7). Or use the keyboard to select the compound that you want to add and click on the plus sign next to the name of the compounds.

**Figure 11.7:** Adding the list of compounds to generate a simulated spectrum.

- In this example, we will select all seven compounds that we have previously added to the list. Before we generate the data set, we need to set the number of classes that we want to simulate in the data set using the middle top panel under the “General settings” (green box, Figure 11.8).

**Figure 11.8:** General settings can be used to set the number of classes, resolution, and the distribution of the simulated dataset.

- Insert the number of classes in the dataset as 2 and change the default number of points in spectra (resolution) from 1,024 to 4,096. The resolution can be changed to higher or lower values as appropriate. Set the minimum and maximum values of the mean as well as the range of standard deviations that you want for your chosen compounds here. An example is shown in Figure 11.9

**Figure 11.9:** An example of the settings that have been selected.

- To select which compounds in this list are considered biomarkers in the data set, tick the selected compound, under the column heading of biomarkers, as shown in Figure 11.10. In this example, four compounds have been chosen as biomarkers as indicated by the green ticks against them.

**Figure 11.10:** Compounds selected as biomarkers are indicated by green ticks in the biomarker column.

- Next the user can specify the number of spectra they want to represent each class, the concentration, as well as the standard deviation, for each biomarker in each class. An example of these parameter values is shown in Figure 11.11.

**Figure 11.11:** Parameter settings for selected biomarkers in each class.

- To generate a simulated data set using the values that you have defined, click on the “Generate dataset” tab on the top middle section of the page, Figure 11.12.

**Figure 11.12:** Use the “Generate dataset” tab to generate the simulated dataset using the parameter settings.

- The simulated spectral data set containing two classes of spectral data is shown: Class 1 in red, and class 2 in cyan, Figure 11.13. To download the simulated data set that you have created, click on the download arrow (red circle, Figure 11.3). All the features, such as box plot, tracking info, or calculate STOCSY, are available as shown in previous videos. Please refer to previous videos on how to do this.

**Figure 11.13:** Simulated dataset containing two classes.

**References**

Blaise, B.J.*, et al.* Power Analysis and Sample Size Determination in Metabolic Phenotyping. *Anal Chem* 2016;88(10):5179-5188.

Bylesjo, M.*, et al.* OPLS discriminant analysis: combining the strengths of PLS-DA and SIMCA classification. *Journal of Chemometrics* 2006;20:341-351.

Cloarec, O.*, et al.* Statistical total correlation spectroscopy: an exploratory approach for latent biomarker identification from metabolic 1H NMR data sets. *Anal Chem* 2005;77(5):1282-1289.

Fatemi, N.*, et al.* DNA methylation biomarkers in colorectal cancer: Clinical applications for precision medicine. *Int J Cancer* 2022;151(12):2068-2081.

Holmes, E.*, et al.* Incomplete Systemic Recovery and Metabolic Phenoreversion in Post-Acute-Phase Nonhospitalized COVID-19 Patients: Implications for Assessment of Post-Acute COVID-19 Syndrome. *J Proteome Res* 2021;20(6):3315-3329.

Hood, L. and Flores, M. A personal view on systems medicine and the emergence of proactive P4 medicine: predictive, preventive, personalized and participatory. *N Biotechnol* 2012;29(6):613-624.

Jolliffe, I.T. and Cadima, J. Principal component analysis: a review and recent developments. *Philos Trans A Math Phys Eng Sci* 2016;374(2065):20150202.

Li, J.V.*, et al.* Metabolic surgery profoundly influences gut microbial-host metabolic cross-talk. *Gut* 2011;60(9):1214-1223.

Li, J.V.*, et al.* Roux-en-Y gastric bypass-induced bacterial perturbation contributes to altered host-bacterial co-metabolic phenotype. *Microbiome* 2021;9(1):139.

Loo, R.L.*, et al.* Strategy for improved characterization of human metabolic phenotypes using a COmbined Multi-block Principal components Analysis with Statistical Spectroscopy (COMPASS). *Bioinformatics* 2021;36(21):5229-5236.

Norman, P.E.*, et al.* Cohort Profile: The Health In Men Study (HIMS). *Int J Epidemiol* 2009;38(1):48-52.

Perez-Diaz-Del-Campo, N.*, et al.* Diagnostic scores and scales for appraising Nonalcoholic fatty liver disease and omics perspectives for precision medicine. *Curr Opin Clin Nutr Metab Care* 2022;25(5):285-291.

Sarafian, M.H.*, et al.* Bile acid profiling and quantification in biofluids using ultra-performance liquid chromatography tandem mass spectrometry. *Anal Chem* 2015;87(19):9662-9670.

Seyfried, F.*, et al.* Urinary phenotyping indicates weight loss-independent metabolic effects of Roux-en-Y gastric bypass in mice. *J Proteome Res* 2013;12(3):1245-1253.

Stamler, J.*, et al.* INTERMAP: background, aims, design, methods, and descriptive statistics (nondietary). *J Hum Hypertens* 2003;17(9):591-608.

Torell, F., Skotare, T. and Trygg, J. Application of Multiblock Analysis on Small Metabolomic Multi-Tissue Dataset. *Metabolites* 2020;10(7).

Yeap, B.B.*, et al.* Progressive impairment of testicular endocrine function in ageing men: Testosterone and dihydrotestosterone decrease, and luteinizing hormone increases, in men transitioning from the 8th to 9th decades of life. *Clin Endocrinol (Oxf)* 2018;88(1):88-95.
